# Food Monitoring: Limitations of Accelerated Storage to Predict Molecular Changes in Hazelnuts (*Corylus avellana* L.) under Realistic Conditions Using UPLC-ESI-IM-QTOF-MS

**DOI:** 10.3390/metabo13101031

**Published:** 2023-09-25

**Authors:** Henri Loesel, Navid Shakiba, Soeren Wenck, Phat Le Tan, Tim-Oliver Karstens, Marina Creydt, Stephan Seifert, Thomas Hackl, Markus Fischer

**Affiliations:** 1 Hamburg School of Food Science, Institute of Food Chemistry, University of Hamburg, Grindelallee 117, 20146 Hamburg, Germany; henri.loesel@uni-hamburg.de (H.L.); navid.shakiba@uni-hamburg.de (N.S.); soeren.wenck@uni-hamburg.de (S.W.); phat.le.tan@studium.uni-hamburg.de (P.L.T.); tim-oliver.karstens@studium.uni-hamburg.de (T.-O.K.); marina.creydt@uni-hamburg.de (M.C.); stephan.seifert@uni-hamburg.de (S.S.); thomas.hackl@uni-hamburg.de (T.H.); 2 Institute of Organic Chemistry, University of Hamburg, Martin-Luther-King-Platz 6, 20146 Hamburg, Germany

**Keywords:** hazelnut, storage, ion mobility, mass spectrometry, *Corylus avellana*, antioxidative capacity

## Abstract

Accelerated storage is routinely used with pharmaceuticals to predict stability and degradation patterns over time. The aim of this is to assess the shelf life and quality under harsher conditions, providing crucial insights into their long-term stability and potential storage issues. This study explores the potential of transferring this approach to food matrices for shelf-life estimation. Therefore, hazelnuts were stored under accelerated short-term and realistic long-term conditions. Subsequently, they were analyzed with high resolution mass spectrometry, focusing on the lipid profile. LC-MS analysis has shown that many unique processes take place under accelerated conditions that do not occur or occur much more slowly under realistic conditions. This mainly involved the degradation of membrane lipids such as phospholipids, ceramides, and digalactosyldiacylglycerides, while oxidation processes occurred at different rates in both conditions. It can be concluded that a food matrix is far too complex and heterogeneous compared to pharmaceuticals, so that many more processes take place during accelerated storage, which is why the results cannot be used to predict molecular changes in hazelnuts stored under realistic conditions.

## 1. Introduction

Hazelnuts (*Corylus avellana* L.) represent an important raw material in the confectionery industry. They are used in chocolate, spreads, and pastries, as well as in-shell for direct consumption. Since hazelnuts are harvested seasonally but demand exists throughout the year, long storage periods are inevitable, which can lead to significant quality deficiencies [1,2,3]. These deficiencies can become apparent through a bitter off-taste, rancidity, or an infestation with microorganisms [4,5,6]. Depending on storage conditions and duration, this may not always be the case, and methods for testing for rancidity or microbial contamination are not necessarily suitable for detecting the aging process [7,8,9]. Weak harvest years pose a particular problem in meeting the demand for hazelnuts. In 2014, for example, the harvest in Turkey was poor due to hail and frost, resulting in a global price increase [10]. In order to meet the demand in such a low-yield year, there is a presumption that new crops are mixed with old crops. To analyze a long storage process in detail, accelerated storage under harsher conditions (e.g., increased temperature and humidity) is performed, especially for pharmaceuticals. The aim of this is to analyze stability and degradation products in a shorter time to determine shelf-life and to draw conclusions about potential storage issues [11,12].

Numerous studies have already been carried out that have investigated the quality of hazelnuts or hazelnut oil under a wide variety of storage conditions, mainly analyzing classical quality parameters such as peroxide value, total oxidation value, antioxidative capacity, or sensory parameters [4,9,13,14,15,16,17]. Hazelnuts stored under mild conditions (e.g., low temperature, low humidity, or modified atmosphere) showed minor changes compared to samples before storage or samples stored under harsher conditions [4,9]. This shows that the aging process is complex and should be analyzed in more detail. Therefore, the use of non-targeted analysis is particularly advantageous in such research areas since high resolution instrumental approaches are capable of detecting up to several thousand metabolites in a single measurement [18]. Nevertheless, there are only a few studies that have focused on the storage of hazelnuts. Two studies employed two-dimensional GC-MS analysis to identify storage-related substances like 2-octanol, linked to lipid oxidation [19], and marker substances, including octanoic acid and short-chain lactones like γ-nonalactone, associated with sensory characteristics like moldiness or rancidity [20]. Additionally, autoxidation products (hexanal, heptanal, octanal, and nonanal) were investigated using GC-MS to analyze their impact on hazelnut quality and rancidity [21]. Two studies were published investigating the changes of hazelnut oil during storage by LC-MS. They showed that triacylglycerols (TAGs) and phospholipids, among others, are degraded, while several oxidized lipids are formed under accelerated conditions [22,23]. In addition, the analysis of the autoxidation of cold-pressed oil at accelerated conditions (60 °C) showed that linoleic acid and sphingolipid metabolism are affected by storage [23]. However, a comparison of molecular changes under different storage conditions has not yet been carried out in detail—especially the transferability of results from accelerated short-term storage to realistic long-term storage. Studies comparing both storage approaches have so far mainly focused on sensory properties [24,25].

Therefore, the aim of this study was to investigate and compare metabolic processes in hazelnuts under different storage conditions. For this, six hazelnut samples from the 2018 and 2019 crop years were stored under accelerated short-term and realistic long-term conditions. Based on this, it should be analyzed whether accelerated storage experiments can be used for the analysis of foods with low water content, although foods are characterized by a much more complex matrix than pharmaceuticals. This means that more reactions and interactions between ingredients are likely. For this purpose, in the present study, the hazelnut samples before and after storage were analyzed by UPLC-ESI-IM-qToF-MS (ultrahigh performance liquid chromatography-electrospray ionization-ion mobility-quadrupole time of flight-mass spectrometry), focusing on the lipid profile. An ion mobility cell was used to separate isobaric compounds, providing the collision cross section (CCS) as an additional parameter for metabolite identification [26]. In addition, the antioxidant capacity of the samples was determined to compare the potential of molecular fingerprinting analyses, which encompass a wide range of metabolites, with a classical parameter for the analysis of aging processes.

## 2. Materials and Methods

### 2.1. Reagents and Chemicals

Ultrapure water was obtained by purifying demineralized water in a Direct-Q 3 UV-R system (Merck, Millipore, Darmstadt, Germany). LC-MS-grade acetonitrile, methanol, ammonium formate, and HPLC-grade chloroform were purchased from Carl Roth GmbH and Co. KG (Karlsruhe, Germany). LC-MS-grade isopropanol was from Merck KgaA (Darmstadt, Germany). LC-MS tuning mix and hexamethoxyphosphazine, used as tuning standards, as well as hexakis(1H,1H,3H-tetrafluoropropoxy)phosphazine, and purine, used as lock masses, were purchased from Agilent Technologies (City of Santa Clara, CA, USA). Acetic acid, ethanol, sodium carbonate, disodium hydrogen phosphate, sodium chloride, potassium chloride, and potassium dihydrogen phosphate (all analytical-grade) were from Carl Roth GmbH and Co. KG (Karlsruhe, Germany). 2,2’-Azino-bis-(3-ethylbenzothiazoline-6-sulfonic acid) diammonium salt (ABTS), fluorescein, and trolox (all analytical-grade) were from Sigma-Aldrich Chemie GmbH (Deisenhofen, Germany). 2,2’-Azobis(2-methylpropionamidine) dihydrochloride (AAPH), potassium peroxodisulphate, and gallic acid (both of analytical-grade) were purchased from Fisher Scientific GmbH (Schwerte, Germany). Folin-Ciocalteu phenol reagent (analytical-grade) was obtained from Merck KgaA (Darmstadt, Germany). Acetone (analytical-grade) was obtained from VWR International LLC (Fontenay-sous-Bois, France).

### 2.2. Samples and Storage Conditions

Samples were provided by external project partners from the confectionery industry. Three samples from the 2018 and three from the 2019 crop years were investigated (corresponding to six biological replicates). They were mixtures of varieties that were grown in Turkey. The hazelnuts were ≥ 11 mm in diameter and cracked before storage. When choosing the storage conditions, a distinction was made between accelerated short-term and realistic long-term storage. Table 1 and Table S1 show the storage conditions applied, and an overview of the samples, respectively.

### 2.3. Sample Preparation

For the analysis of samples exposed to accelerated short-term storage, aliquots (100 g) of each of the six samples were taken after two and four days, as well as every second week until week twelve and every fourth week thereafter (corresponds to eleven stored time points for each of the six samples per storage condition). For the analysis of samples exposed to realistic long-term storage, a sample was aliquoted every 3 months (corresponds to six stored time points for each of the six samples per storage condition). An overview of the storage durations can be found in Table S2 in the Supplementary Materials. After aliquoting, the samples were snap frozen in liquid nitrogen and stored at −80 °C. For analysis, they were ground under dry ice cooling (1/2 *wt*/*wt*, hazelnut/dry ice) using a knife mill (Grindomix GM 300, Retsch, Haan, Germany) and 6.0 ± 0.1 g of the homogenized powder was lyophilized (Beta 1–8 LSCplus, Martin Christ Freeze Dryers GmbH, Osterode, Germany) over a period of 24 h, stirring once after half the time had elapsed to ensure uniform drying.

### 2.4. UPLC-ESI-IM-QTOF-MS Analysis

Metabolites were extracted using a mixture of chloroform/isopropanol (1:2, *v*/*v*) [27]. For this purpose, 50.0 ± 0.5 mg was mixed with 1 mL of the extraction solvent and two steel balls (⌀ = 3 mm) in a 1.5 mL reaction tube (Eppendorf, Hamburg, Germany). Subsequently, the samples were ball milled for 3 min at 3 m/s using a Bead Ruptor 24 equipped with a 1.5 mL microtube carriage kit (Biolabproducts, Bebensee, Germany). The suspension obtained was centrifuged for 5 min at 14,000× *g* and 4 °C (Sigma, Osterode, Germany). The resulting supernatant was centrifuged again for 20 min at the same conditions, to ensure that proteins were completely removed. For analysis, the supernatant was diluted 1:2 (*v*/*v*) using the extraction solvent. One analytical replicate was analyzed for each sample.

A 6560 Ion Mobility qToF LC-MS system (Agilent Technologies, City of Santa Clara, CA, USA) was used for the analysis of the extracts. Chromatographic separation was performed on an Agilent UPLC system (1290 Infinity II, Agilent Technologies) equipped with a high-speed pump (G7120A, 1290 high-speed pump), a multisampler (G7167B, 1290 multisampler), and a temperature-controlled column compartment (G7116B, 1290 MCT). For chromatographic separation, an Accucore RP-MS UPLC column (150 mm × 2.1 mm i.d., 2.6 μm) was used, equipped with a guard column of the same material (10 mm × 2.1 mm, 2.6 μm) (Thermo Fisher Scientific, Braunschweig, Germany). 

The samples were measured in a randomized order to balance instrumental drifts. To check the reproducibility of the instrument, a quality control (QC) sample (native hazelnut sample) was injected every ten measurements. Furthermore, blank samples were measured every five samples where no injection was done. To ensure that signals identified in hazelnut samples were not due to the contamination of the instrument, chemicals, or consumables, an extraction blank was also analyzed analogously. The column oven was tempered to 25 °C to ensure constant conditions, with a flow rate of 400 μL/min. The mobile phase used ultra-purified water (A) and acetonitrile/isopropanol (B; 1:2, *v*/*v*), both with a concentration of 0.1 mmol/L ammonium formate buffer at pH 3.5. The gradient started with 65% of B for 2 min, increased to 85% of B within 3 min, which was subsequently kept constant for 1 min, and afterward raised to 95% of B by minute 22. Subsequently, it was switched to 100% B within 1 min and held constant for 2 min. Finally, re-equilibration was performed for 5 min. For all samples, the injection volume was set to 2 μL.

The samples were measured in positive ionization mode in MS^1^ mode in a mass range from 100–1700 *m*/*z* with the following mass spectrometer settings: gas temperature 225 °C, drying gas flow rate 10 L/min, nebulizer 40 psi, sheath gas temperature 375 °C, sheath gas flow rate 12 L/min, capillary voltage 3000 V. The MS/MS mode with collision energies of 10 eV, 20 eV, and 40 eV was used for the identification of possible marker substances. 

The calibration of the instrument was made externally with a mixture of hexamethoxyphosphazine and a tuning mix (Agilent Technologies, City of Santa Clara, CA, USA, part number: G1969-85000) immediately before the series of measurements in low mass range mode (*m*/*z*: 50–1700) and extended dynamic range mode. In addition, a lock mass calibration with purine and hexakis(1H,1H,3H-tetrafluoropropoxy)phosphazine was carried out using a second sprayer during the measurements.

Furthermore, additional measurements were performed in IM-ToF mode to determine CCS values with the following parameters: drift gas nitrogen; drift gas pressure 3.95 Torr; frame rate 1 frame/s; IM transient rate 16 IM transients/frame; max. drift time 60 ms; trap fill time 3900 μs; trap release time 250 μs; multiplexing pulse sequence length 4 bit; drift tube entrance 1700 V; drift tube exit voltage 250 V; rear funnel entrance voltage 240 V; rear funnel exit voltage 43 V. Drift times were calibrated by infusing the Agilent Technologies ESI tune mix under the same conditions for 1 min.

### 2.5. Method for Extracting Antioxidative Compounds

To extract antioxidative compounds, 50 to 60 mg of the samples were suspended with 1 mL of extracting solvent (acetone/water (7:3, *v*/*v*) with an addition of 0.1% (*v*/*v*) acetic acid) und two steel balls (⌀ = 3.25 mm) in a 1.5 mL reaction tube (Eppendorf, Hamburg, Germany). Subsequently, the samples were ball milled for 3 min at 3 m/s using a Bead Ruptor 24 equipped with a 1.5 mL microtube carriage kit (Biolabproducts, Bebensee, Germany). The remaining suspension was centrifuged for 5 min at 14,000× *g* and 4 °C. Subsequently, the supernatant was analyzed.

### 2.6. Analysis of the Antioxidant Capacity Using the Trolox Equivalent Antioxidant Capacity Assay (TEAC)

The TEAC assay was performed according to another study with slight variations [28]. Measurements were carried out on a SpectraMax M5e instrument, which was controlled using the SoftMax Pro 6.1 software (Molecular Devices, LCC, San Jose, CA, USA). First, the ABTS radical was prepared by mixing ABTS (38.40 mg in 10 mL water) and potassium peroxodisulphate (6.62 mg in 10 mL water) and then stored in the absence of light at room temperature for 24 h [29]. Immediately before measurement, this solution was diluted with phosphate buffer (1.42 g disodium hydrogen phosphate, 0.27 g potassium dihydrogen phosphate, 8.00 g sodium chloride, and 0.20 g potassium chloride in 1 L water, pH 7.4) to an absorbance of 0.7 ± 0.1 at 730 nm. Subsequently, 20 μL of a sample extract, standard, or blank was added to a 96-well microtiter plate and mixed with 200 μL of the diluted ABTS solution. After incubation for 10 min in the dark at 22 °C, the absorbance was measured at 730 nm. Calibration was performed using a Trolox calibration and results were reported as Trolox equivalents (TAE) per 100 g sample (mmol TAE/100 g). All measurements were performed at 22 °C. 

### 2.7. Analysis of the Antioxidant Capacity Using the Oxygen Radical Absorbance Capacity Assay (ORAC)

The ORAC assay was performed according to a protocol previously published elsewhere, with minor modifications [28]. A total of 10 μL of a sample extract, standard, or blank was added to a 96-well microtiter plate and mixed with 100 μL phosphate-buffer (1.42 g disodium hydrogen phosphate, 0.27 g potassium dihydrogen phosphate, 8.00 g sodium chloride, and 0.20 g potassium chloride in 1 L water, pH 7.4) and 25 μL of a fluorescein solution (12 μmol/L) in phosphate buffer. After the reaction mixture was incubated at 37 °C for 10 min, 150 μL AAPH (130 mM in phosphate-buffer) was added to induce radical formation [30]. The reaction progress was monitored at 37 °C by measuring the fluorescence of fluorescein until no fluorescence was measurable (excitation: 485 nm, emission: 520 nm). Calibration was performed using a Trolox calibration series and results were reported as mmol TAE/100 g. 

### 2.8. Determination of the Total Phenolic Content According to Folin-Ciocalteu

The Folin-Ciocalteu method was performed according to the protocol of another study, with minor adjustments [28]. A 20 μL quantity of a sample extract, standard, or blank was added to a 96-well microtiter plate and mixed with 75 μL of sodium-carbonate-buffer (18.76 g sodium-carbonate in 250 mL water). Then, 100 μL freshly diluted Folin-Ciocalteu-solution (1:10, *v*/*v*) was added and incubated for two hours at room temperature in the dark. The measurement was performed at 22 °C at a wavelength of 740 nm. Calibration was carried out using a gallic acid calibration series and results were reported as gallic acid equivalents (GAE) per 100 g sample (mmol GAE/100 g). 

### 2.9. Data Processing and Chemometrics

Feature detection was carried out with the MS^1^ data using the Qualitative Analysis 10.0 software (Agilent Technologies, City of Santa Clara, CA, USA) with the following parameters: restrict retention time to 0.2–30.0 min; ion intensity ≥ 100 counts; allowed ion species +H, +Na, +K, +NH_4_; limit assigned charge states to a range of 1–2. The data were exported to Mass Profiler Professional 15.1 (Agilent Technologies, City of Santa Clara, CA, USA) as .cef files and time as well as mass alignment were performed using the following parameters: Perform RT correction checked; maximum allowed RT shift 0.4% ± 0.4 min; mass window 10.0 ppm ± 10.0 mDa. One feature had to be detectable in at least 50% of the samples of one storage condition. Missing values were replaced with the value 1. Furthermore, the data were vector normalized. Occurring batch effects were removed by autoscaling each batch separately, a well-known batch standardization technique, which we have already applied as well (See Figures S1 and S2 for more information) [31,32].

CCS values were determined by first demultiplexing measurements taken in IM-ToF mode using PNNL PreProcessor software (version 2020.03.23). The following parameters were selected: demultiplexing checked; moving average window size 5 frame; moving average smoothing checked; *m*/*z* not used; drift 3; chromatography/infusion 3, signal intensity lower threshold 20 counts. Subsequently, the CCS values were calibrated using the IM-MS Browser software (version 10.0, Agilent Technologies). Finally, four-dimensional feature finding was performed using Mass Profiler software (version 10.0, Agilent Technologies). The parameters were: restrict RT to 0.0–30.0 min; ion intensity >150.0 counts; isotope model common organic (no halogens); limit charge states to a range of 1–2; report single-ion features with charge state z = 1; RT tolerance = ±10.0% + 0.50 min; DT tolerance = ±1.5%; mass tolerance = ±20.0 ppm + 2.0 mDa; Q-Score > 70.0.

MATLAB R2021b (The MathWorks Inc., Natick, MA, USA) was used for principal component analysis (PCA) as well as to train and validate classification models using the linear support vector machine algorithm (SVM) from the Classification Learner app with the MS^1^ data. The samples of each storage condition were divided into pre- and post-storage (defined as: ST1–ST3 ≥ 12 weeks; LT1 and LT2 ≥ 12 months). During training, data from one of the six samples were omitted completely and then used for testing the trained model. This process was repeated for all six samples, resulting in six test and training sets. In accelerated short-term storage, a test set contained one pre-stored sample and four post-stored samples (12, 16, 20, and 24 weeks), while one pre-stored sample and three post-stored samples (12, 15, and 18 months) were in one test set during realistic long-term storage. 

The R package Pomona (Version 1.0.1, https://github.com/silkeszy/Pomona (accessed on 23 October 2022)) was used for Boruta feature selection with the MS^1^ data with the parameters: ntree = 10,000, min.node.size = 1, mtry = 142, importance = impurity_corrected, maxRuns = 100, and *p* Value = 0.01 [33].

The MS^1^ data were used for the characterization of metabolites selected by Boruta (high resolution mass, retention time, CCS-value). The high resolution mass was used to predict possible chemical formulas with calculated mass deviations <5 ppm. Furthermore, retention time represented another characteristic. Structural information was obtained from the MS/MS spectra. In addition, identification suggestions were compared using the CCS value with the LipidCCS database [34]. For the substances for which a database entry was available, the experimental mass deviated by a maximum of 1.2%.

Differences between pre- and post-stored samples in the TEAC, ORAC, and Folin-Ciocalteu assays were determined using the analysis of variance (ANOVA) single factor analysis tool in Excel 2019 (Microsoft, WA, Redmond, USA), with a significant difference at a *p*-value of ≤0.01.

## 3. Results and Discussion

### 3.1. Principal Component Analysis (PCA) for Evaluation of the Greatest Variances and the Applicability of the Data Processing

The aim of this study was to investigate and compare metabolic processes in hazelnuts under different storage conditions. Furthermore, it should be concluded whether results from accelerated short-term storage are transferable to realistic long-term storage.

**Figure 1:**
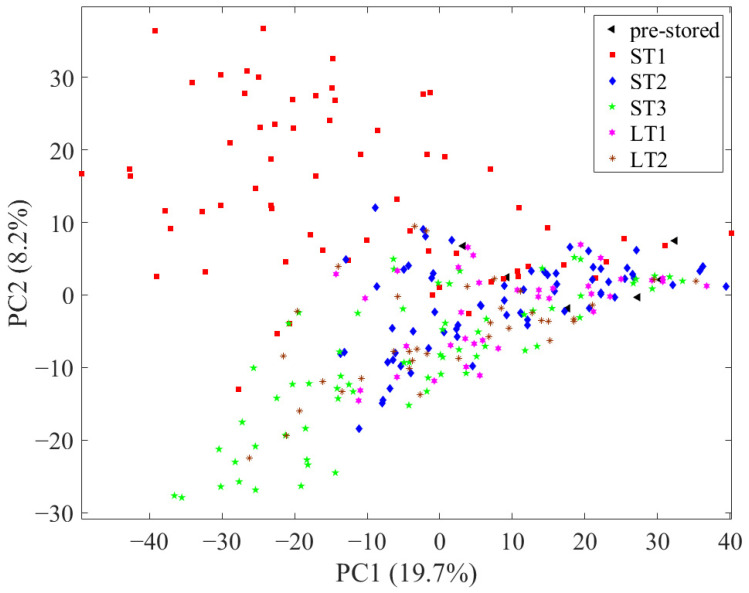
Scores of the principal component analysis using all 1875 variables with samples depicted according to their storage condition.

First, to assess the reproducibility of the LC-MS system, all measurements, including the 32 measurements of the QC sample, were evaluated with PCA, with all 1875 variables after pre-processing (see Section 2.5. in Material and Methods and Figures S1 and S2 in the Supplementary Materials). An exemplary total ion chromatogram of a hazelnut measurement is shown in Figure S3. In the scores, the measurements of the QC samples showed only a little scatter, so the data were considered comparable. Subsequently, another PCA was performed with all 1875 variables without the QC sample measurements, in which the samples were depicted according to their storage condition (Figure 1). In this, the pre-stored samples (black triangles) are in the middle on the right side of the principal component (PC) 1 and the post-stored samples scatter to the left side of PC1. In this context, the scattering implies the degree to which the chemical profile of the pre- and post-stored samples differs. In the scores, the samples stored under ST1 (red squares; 40 °C; 75% rel. humidity) and ST3 (green pentagrams; 40 °C; 25% rel. humidity) conditions spread the most from the pre-stored samples, which is an indication that higher temperatures lead to increased changes in the metabolome. In addition, samples stored under ST1 conditions scatter to the upper left and samples stored under ST3 conditions to the lower left, implying that different metabolic processes occurred in both storage conditions. The samples of storage condition ST2 (blue diamonds; 25 °C; 60% rel. humidity) and LT1 (pink hexagrams; 10 °C; 75% rel. humidity) overlap considerably and are comparatively close to the pre-stored samples. Therefore, it can be concluded that there are comparatively few storage-related differences in the chemical profile in samples stored under these conditions. It can be seen that the development of storage-induced changes in the nonpolar metabolome is comparable for samples stored at 25 °C within a time period of 24 weeks (ST2) and samples stored at 10 °C within a time period of 18 months (LT1). Compared to this, the samples that have been stored at 19 °C (LT2; brown stars) show more pronounced differences, as they are scattered further away from the pre-stored samples. 

In summary, the unsupervised data analysis with PCA shows that the degree of storage-related differences depends on the combination of storage condition and storage duration. In the following, these differences are investigated in more detail using supervised methods.

### 3.2. Classification of Pre- and Post-Stored Samples 

To evaluate the capability of the LC-MS approach to identify hazelnuts that have been stored for an extended period and to determine the degree of storage-related differences, SVM-based classification models were trained and tested. The classification models were built separately for each storage condition, distinguishing between pre-stored and post-stored samples by leave-one-sample-out cross validation. The classification results obtained are shown in Table 2. An overview of the training set results as well as the specification of the misclassified samples during validation can be found in Table S3 in the Supplementary Materials.

The classification results show that for ST1 and ST3 (40 °C with 75% and 25% rel. humidity), only one misclassification was observed, while for condition ST2 (25 °C; 60% rel. humidity), the pre-stored samples from five sets were incorrectly predicted. Hence, higher temperatures at accelerated short-term storage led to the successful separation of pre- and post-stored samples. In contrast, three of six pre-stored samples were correctly predicted for the realistic long-term storage condition LT2 (19 °C; 50% rel. humidity). Since LT2 and ST2 are quite similar except for the storage time, the changes occurring in LT2 must develop comparatively slowly. At low temperature and long storage time (LT1; 10 °C; 75% rel. humidity; 18 months), the nonpolar metabolome cannot be used to reliably differentiate between pre- and post-stored samples, since five pre-stored samples were predicted to be post-stored. This probably correlates with the fact that the growth of microorganisms, as well as enzymatic and non-enzymatic reactions, are slowed down at these temperatures [35,36,37]. In summary, the results show that the success of the detection of stored samples is highly dependent on the storage conditions, especially the temperature. Furthermore, this supports previous observations from the analysis with PCA that storage-related differences are differentially pronounced depending on the storage condition.

### 3.3. Comparison of Selected Features for the Different Storage Conditions

**Figure 2:**
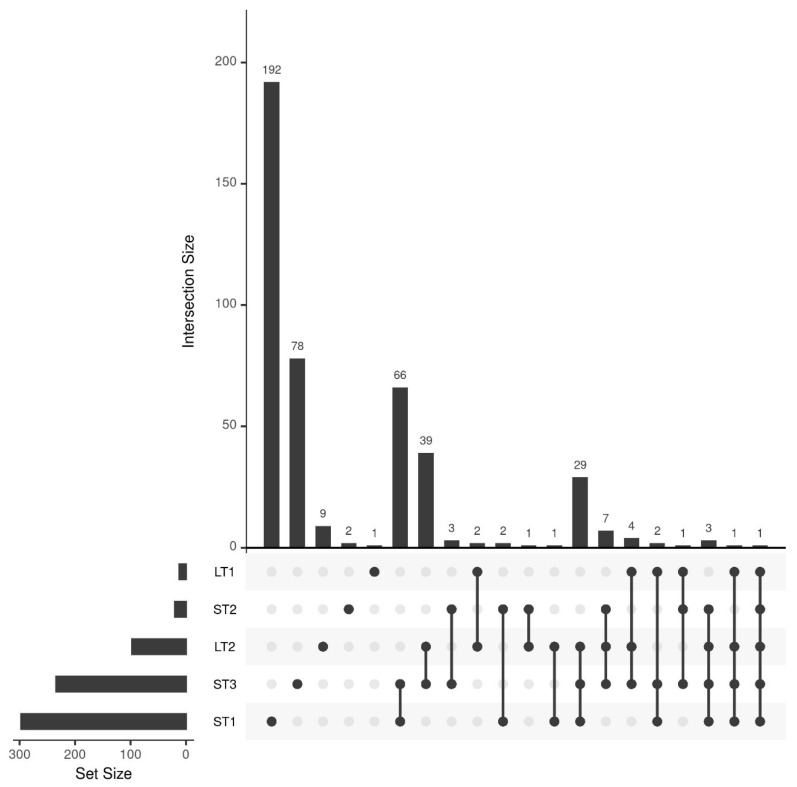
Upset plot showing the number of selected features of the different storage conditions and their overlap. A total of 297, 20, 234, 12, and 97 features were selected for ST1, ST2, ST3, LT1, and LT2, respectively.

The classification results indicated that different metabolomic changes occur in hazelnuts when they are stored differently. To comprehensively analyze these changes between pre- and post-stored samples, the multivariate feature-selection approach Boruta was applied, which assesses variable importance by comparing features to randomized shadow variables [38]. Figure 2 summarizes the results in an upset plot. In addition, a comprehensive list of identified features can be found in Table S4 of the Supplementary Materials.

The most features were selected for ST1 (297 features) and ST3 (234 features), confirming the conclusions of the previous section that storage under high temperature causes large differences. The overlap between the selected features of those two storage conditions is also comparatively large (66 features), whereas many unique metabolites were selected for ST1 (192 features). This shows that the combination of high temperature and humidity leads to unique, large changes in the metabolome. The third most features, namely 97, were selected for LT2 (19 °C; 50% rel. humidity). In contrast, only 20 features were selected for the samples of ST2 (25 °C; 60% rel. humidity). Since these samples were stored under comparable conditions to LT2 for a shorter time, it is evident that at temperatures around 20 °C, storage-related changes only occur after an extended storage period, which we had already concluded from the classification results in Section 3.2. The overlap of LT2 and ST3 with 39 features, as well as with ST1 and ST3 with 29 features, is comparably high, indicating that some changes that occur more rapidly at elevated temperature also occur after an extended storage period at milder conditions. For LT1, the samples were stored at a comparatively mild temperature and a high humidity (10 °C and 75% rel. humidity), only twelve features were selected, suggesting that elevated humidity alone does not significantly affect metabolic changes.

The feature selection results are generally in agreement with the classification results from the previous section, since the conditions that showed separation between pre- and post-storage (ST1, ST3, and LT2) also have high numbers of selected features. However, the analysis of the selected features also shows that accelerated food storage experiments cannot be used for shelf-life studies like in the pharmaceutical industry [11]. The reason for this is that in accelerated short-term storage, many features are selected that do not occur in realistic long-term storage under moderate environmental conditions.

### 3.4. Analysis and Comparison of Storage-Induced Changes in the Metabolome of Hazelnuts Stored under Accelerated and Realistic Conditions

In the following section, the selected metabolites and the metabolic changes that occurred under different storage conditions are analyzed in more detail. The aim is to compare storage-induced changes under different storage conditions and to evaluate to what extent the accelerated short-term storage can be transferred to realistic long-term storage to predict molecular changes. Therefore, metabolites selected by the Boruta algorithm were identified by their high-resolution mass, MS/MS spectra, and comparison of CCS values with the LipidCCS database (see Table S4 in the Supplementary Materials) [34]. The fragmentation pattern of the discussed substance classes is also explained in the Supplementary Materials [39,40,41,42,43,44,45,46,47].

#### 3.4.1. Analysis of Specific Metabolites for the Accelerated Short-Term Storage Condition with High Temperature (40 °C) and Humidity (75%) (ST1) and Comparison with the other Storage Conditions

As mentioned above, the differences induced by storage at high temperature and humidity (ST1; 40 °C; 75% rel. humidity) are comparatively large and many unique features were identified. This means that processes occurred that did not happen under other conditions. These features were assigned to phospholipids, such as phosphocholines (PC), phosphoethanolamines (PE), and phosphatidylglycerols (PG), ceramides, and digalactosyldiacylglycerides (DGDGs). 

**Figure 3:**
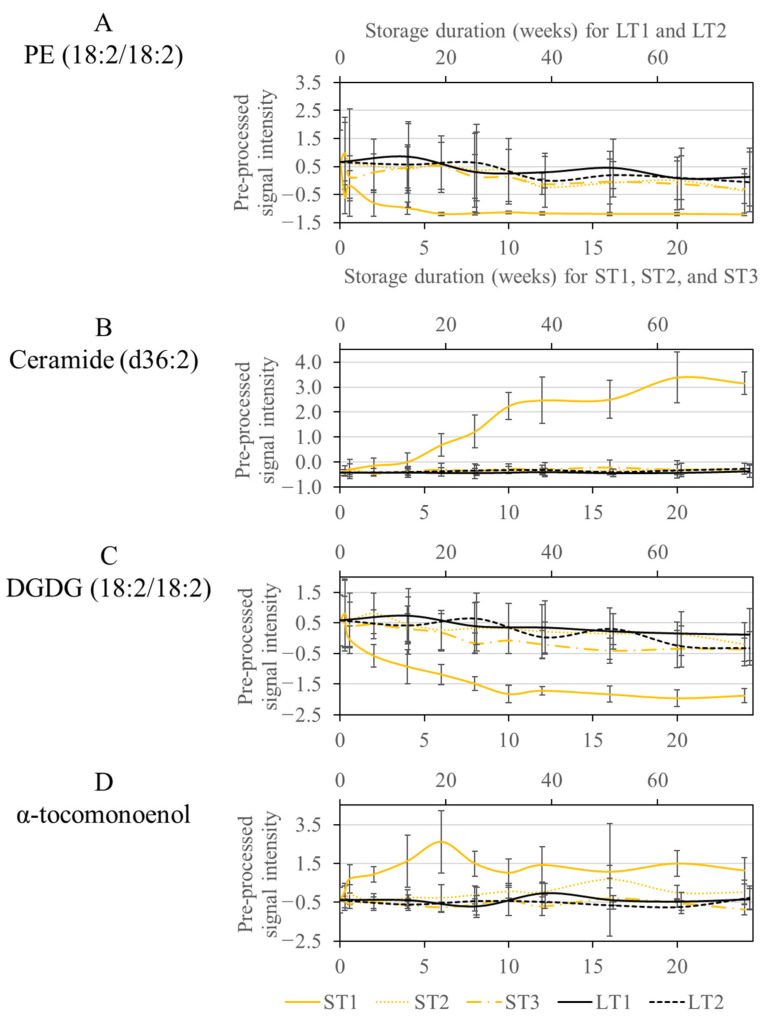
Course of the signal intensities of the metabolites PE (18:2/18:2) (**A**), Ceramide (d36:2) (**B**), DGDG (18:2/18:2) (**C**), and α-tocomonoenol (**D**), which were selected only at the condition ST1. The accelerated short-term storage experiments are shown in yellow (ST1: continuous line, ST2: dotted line, ST3: dash line), while the realistic long-term storage experiments are shown in black (LT1: continuous line, LT2: dotted line). The low horizontal axis belongs to accelerated short-term storage and the upper axis to the realistic long-term storage.

The signal intensities of phospholipids decreased rapidly in samples stored under ST1 conditions during the first five weeks, as shown in Figure 3A, exemplarily for PE (18:2/18:2). Phospholipids were also degraded under the other storage conditions, but not to a large extent, so they were not selected by the Boruta algorithm. It is conceivable that degradation occurs through lipolytic enzymatic activity [48]. Thus, it can be assumed that phospholipases or microorganisms expressing these enzymes are active only at ST1 conditions. Hazelnuts contain hygroscopic constituents such as monosaccharides, which can increase the wet weight at high humidity. An increased water content may support the activity of microorganisms or enzymes. A high temperature (40 °C) in combination with a high humidity (75%) provides a suitable medium for many microorganisms, such as molds, which are probably less able to grow under other storage conditions [6]. 

Another class of compounds, selected only for ST1, were ceramides, which showed an increase in signal intensities, whereas no differences were observed for the other conditions (Figure 3B). Ceramides are a subgroup of sphingolipids, which may be linked to carbohydrates, usually glucose, via an O-glycosidic bond (glycosphingolipids) or to choline by a phosphodiester bond (sphingomyelins) [49]. Enzymatic hydrolysis by phospholipase C to ceramide has already been discussed in the literature [50]. Therefore, it is conceivable that the activity of phospholipases under ST1 conditions could provide unique changes, both for phospholipids and for sphingolipids representing large families of membrane lipids [49]. The involvement of microorganisms is also conceivable, as many microorganisms express phospholipases, which aid in host invasion by disrupting cell membranes [51]. The growth of such microorganisms is supported by elevated humidity and temperature, as is the case for ST1 [6]. The results are in agreement with another study, which also observed the degradation of sphingomyelins during accelerated storage of hazelnut oil [23].

In addition, DGDGs were conspicuous, which were also only selected in ST1. DGDGs are localized in the plastid membrane and are involved in photosynthetic complexes [52]. The concentration decreased exponentially during storage in ST1, with enzymatic degradation by galactolipases being conceivable. Enzymes with galactolipase activity release fatty acids from DGDGs by hydrolysis reactions [53]. In the other storage conditions, DGDGs were also degraded, but so slowly that they were not selected by the Boruta approach (Figure 3C).

Another compound, increased only in ST1, had an *m*/*z* ratio of 429.37 (Figure 3D). The fragment spectrum of this compound showed signals at *m*/*z* 205 and 165 corresponding to known fragments of α-tocopherol (*m*/*z* 431.39 as M + H adduct) (See Figure S4 in the Supplementary Materials). The mass difference of our analysis to α-tocopherol could be explained by an additional double bond. A vitamin E derivative called α-tocomonenol with a double bond in the C11′-position in the isoprenoid side chain has already been described in the literature [54]. The antioxidant capacity of δ-tocomonoenol was investigated in another study and was only slightly lower than that of the saturated forms α- and δ-tocopherol [55]. Other non-antioxidant properties have already been described for α-tocopherol in vitro. Accordingly, it acts as a ligand for proteins and thus also influences gene expression and signal transduction [56]. So far, little is known about these properties of α-tocomonoenol [57]. However, based on its antioxidant properties, α-tocomonoenol could be formed in ST1 as a stress response. Furthermore, other biochemical properties could also be of importance.

#### 3.4.2. Analysis and Comparison of Specific Metabolites Selected for Accelerated Short Term Storage with Increased Temperature (ST1 and ST3) and Realistic Long-Term Storage (LT2)

**Figure 4:**
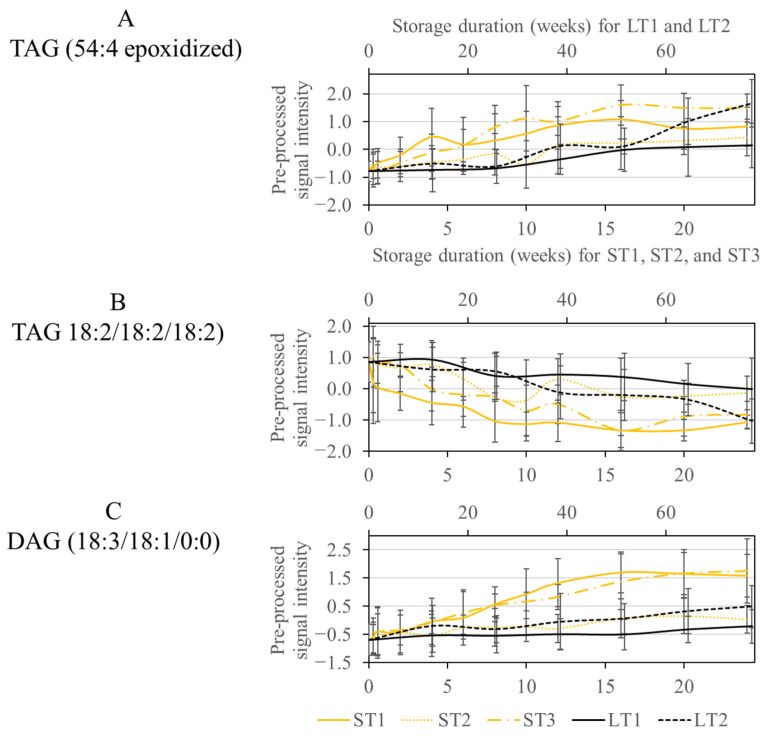
Course of the signal intensity of the metabolites TAG (54:4 epoxidized) (**A**), TAG (18:2/18:2/18:2) (**B**), and DAG (18:3/18:1/0:0) (**C**), from the overlap of the conditions ST1, ST3, and LT2. The accelerated short-term storage experiments are shown in yellow (ST1: continuous line, ST2: dotted line, ST3: dash line), while the realistic long-term storage experiments are shown in black (LT1: continuous line, LT2: dotted line). The low horizontal axis belongs to accelerated short-term storage and the upper axis to the realistic long-term storage.

As discussed previously, various metabolites were selected for LT2 (19 °C; 50% rel. humidity), ST1 (40 °C; 75% rel. humidity), and ST3 (40 °C; 25% rel. humidity). Consequently, some metabolic changes that occurred at high temperature in a short time were also noticeable at low temperature after extended storage (see Section 3.3). The selected features could mainly be assigned to oxidized lipids, more precisely epoxides formed next to the double bond of an unsaturated fatty acid (See Table S4 in the Supplementary Materials). The concentration of these oxylipids increased over the course of storage in all analyzed conditions, but a steeper increase can be observed for ST1, ST3, and LT2 (Figure 4A). For ST1 and ST3, the increase primarily happens in the first weeks, while the realistic long-term storage, LT2, increases more quickly at the end of the storage. The CCS values did not increase due to the oxidation process. However, the retention time changed due to the polar character of the epoxide group, which reduced the affinity to the nonpolar stationary phase and caused the analytes to elute earlier.

Another class of substances selected by the Boruta approach were TAGs with saturated and unsaturated fatty acids, which were degraded due to storage. The selection of TAGs agrees with the selection of oxidized lipids, as these are formed by the degradation of TAGs. This is confirmed because the decrease in TAGs in Figure 4B corresponds to the increase in oxidized lipids in Figure 4A for ST1, ST3, and LT2. 

Another way TAGs are affected by storage is through hydrolysis by lipases [58]. This is a possible reason for selecting diacylglycerides (DAGs), which are formed in a storage-dependent manner, as is most strongly observed at ST1 and ST3 (Figure 4C). Analogous to the lipolytic decrease in phospholipids and DGDGs mentioned above, it is conceivable that lipases are most active under ST1 conditions.

To summarize the results of this section: oxidation processes took place in both accelerated short-term and realistic long-term storage and corresponding metabolites could be identified in all the corresponding storage experiments. Overall, the influence of storage temperature is greater than the influence of storage duration and humidity. That is obvious since metabolic changes were observed at elevated temperatures that were not evident in milder conditions. These changes could be assigned to the degradation of membrane lipids such as phospholipids, sphingolipids, or DGDGs. It can be concluded that accelerated storage conditions catalyze reactions that do not occur at all or only at a very slow rate under realistic conditions. For this reason, it is not possible to transfer the results of accelerated short-term storage to realistic long-term storage.

### 3.5. Determination of the Antioxidant Capacity Using TEAC, ORAC, and Folin-Ciocalteu Assays

The selected features consistent between the accelerated short-term storage conditions ST1 and ST3 and the realistic long-term storage LT2 could be identified as oxidized lipids. Oxidation processes can occur non-enzymatically by lipid peroxidation and enzymatically by lipoxygenases [59,60]. Hazelnut seeds contain several natural antioxidants such as tocopherols, ascorbic acid, or polyphenols, which are supposed to protect oxidation-prone components such as unsaturated fatty acids [61,62]. The increase in oxidized lipids due to storage means that the antioxidant capacity of the samples decreases. This could allow the identification of samples stored incorrectly or for too long based on this parameter. The results of the TEAC, ORAC, and folin-ciocalteu assays are shown in Figures S5–S7 in the Supplementary Materials and an overview of all methods including calculated *p*-values (ANOVA) is in Table S5 in the Supplementary Materials.

Compared to the value for pre-stored samples, AOA decreased under all storage conditions, with the largest differences occurring under accelerated short-term storage conditions with higher temperature (ST1 and ST3), confirming the observations of the LC-MS analysis. In general, the results of all three methods are comparable, but the differences between pre- and post-stored samples were significant (*p*-value ≤ 0.01) only in the folin-ciocalteu assay for the long-term stored samples of ST1 and ST3 (Table S5). The respective analysis of the storage conditions ST2, LT1, and LT2 showed no significant differences (*p*-value > 0.01) even though the obtained mean values are lower. The reason for this is the comparatively high standard deviation of AOA values for pre-stored samples of about 30–50% indicating high biological variance. Overall, the analysis of samples obtained by different storage conditions shows that the determination of the AOA is not sufficient to identify samples that have been stored under high temperatures or long storage periods.

## 4. Conclusions

The aim of this study was to compare molecular changes in hazelnuts stored under accelerated short-term and realistic long-term conditions and to determine whether accelerated storage, which is routinely used in the pharmaceutical industry, can be used as a tool to predict storage-induced chemical changes in food. To detect metabolic changes, UPLC-ESI-IM-qToF-MS analysis was used as a multiparametric method. It was demonstrated that samples stored under elevated temperatures are characterized by more and different metabolic changes than samples that have been stored under moderate conditions. We identified possible marker compounds, such as phospholipids or DGDGs, which are degraded within a few weeks only under accelerated short-term storage conditions. Under both realistic long-term and accelerated short-term storage conditions, the formation of oxidized lipids could be detected, but with different rates of formation. However, determination of the oxidation status by antioxidative capacity analysis is not suitable as an alternative method for the analysis of the aging process, because only minor differences were detected. Furthermore, samples stored at moderate temperatures showed little change in LC-MS analysis even after long storage periods. Overall, high-resolution LC-MS shows great potential for detecting storage-related quality defects in hazelnuts, as samples stored under harsh conditions showed large differences compared to pre-stored or adequately stored samples. Nevertheless, the transferability of accelerated storage experiments from the pharmaceutical industry to food matrices is not possible, since these matrices are too complex and heterogeneous, so that different storage conditions catalyze different processes. For this reason, storage-related differences that occur under harsh conditions cannot be transferred to mild conditions.

## Figures and Tables

**Table 1:** Overview of the different storage conditions characterized by temperature, humidity, and duration, as well as their abbreviations (ST: short-term, LT: long-term).

Abbreviation	Temperature [°C]	Rel. Humidity [%]	Maximum Storage Time
ST1	40	75	24 weeks
ST2	25	60	24 weeks
ST3	40	25	24 weeks
LT1	10	75	18 months
LT2	19	50	18 months

**Table 2:** SVM-based classification results for each storage condition to distinguish between pre- and post-stored samples. The latter are defined by ≥12 weeks (accelerated short-term storage) and ≥12 months (realistic long-term storage).

		Predicted			
			Pre-Stored	Post-Stored	Cohens Kappa
True	ST1	Pre-stored	6	0	1.00
		Post-stored	0	24	
	ST2	Pre-stored	1	5	–0.01
		Post-stored	0	24	
	ST3	Pre-stored	5	1	0.89
		Post-stored	0	24	
	LT1	Pre-stored	1	5	0.14
		Post-stored	1	17	
	LT2	Pre-stored	3	3	0.60
		Post-stored	0	18	

## Data Availability

The LC-MS dataset presented in this study is available on request from the corresponding author. The data is not publicly available due to the data size exceeding the allowable amount of common repositories.

## Supplementary Materials

The following supporting information can be downloaded at: https://www.mdpi.com/article/10.3390/metabo13101031/s1, Figure S1: Scores of the principal component analysis depicted according to distinguish the quality control (QC) samples from the others. The data were previously vector normalized and autoscaled together regarding the two batches. Figure S2: Scores of the principal component analysis depicted according to distinguish the quality control (QC) samples from the others. The data were previously vector normalized and autoscaled separately regarding the two batches. Figure S3: Exemplary TIC of a hazelnut sample with the following assignments of the main substance classes: PC: phosphocholine, PE: phosphoethanolamine, PG: phosphoglycerol, Cer: ceramide, DGDG: digalactosyldiacylglyceride, DAG: diacylglyceride, TAG: triacylglyceride. Figure S4: MS/MS spectrum of the metabolite, which was identified as α-tocomonenol. The collision energy was 10 eV. Figure S5: Bar graph of the results of the TEAC assay. The storage durations of accelerated short-term storage were summarized as follows: short (day 2, day 4, week 2), mid (week 6, week 10, week 12), and long (week 16, week 20, week 24). The storage periods of realistic long-term storage were summarized as follows: short (month 3, month 6), mid (month 9, month 12), and long (month 15, month 18). Figure S6: Bar graph of the results of the ORAC assay. The storage durations of accelerated short-term storage were summarized as follows: short (day 2, day 4, week 2), mid (week 6, week 10, week 12), and long (week 16, week 20, week 24). The storage periods of realistic long-term storage were summarized as follows: short (month 3, month 6), mid (month 9, month 12), and long (month 15, month 18). Table S1: Overview about the metadata of the stored hazelnut samples. Table S2: Overview about the chosen storage durations. Table S3: Results of the classification of pre- and post-storage samples (post storage is defined by accelerated short-term storage ≥12 weeks and realistic long-term storage: ≥12 months; T_0_: pre-storage, M_12_: 12 months). The Sample-ID column shows which sample was not included in the training set and was subsequently classified using the trained model. Identification parameters; Table S4: Summary of identification parameters of marker compounds that are affected by storage. Table S5: Results from the determination of antioxidant capacity (AOA) with the TEAC, ORAC, and Folin-Ciocalteu (FC) assays. The storage durations of accelerated short-term storage were summarized as follows: short (day 2, day 4, week 2), mid (week 6, week 10, week 12), and long (week 16, week 20, week 24). The storage periods of realistic long-term storage were summarized as follows: short (month 3, month 6), mid (month 9, month 12), and long (month 15, month 18).
