# Supplementary material for: Food Monitoring: Limitations of Accelerated Storage to Predict Molecular Changes in Hazelnuts (Corylus avellana L.) under Realistic Conditions Using UPLC-ESI-IM-QTOF-MS
Source: Metabolites. 2023 Sep 25;13(10):1031. doi: 10.3390/metabo13101031 (PMC10608644; doi:10.3390/metabo13101031)
Supplement: Supplementary file 1 [file metabolites-13-01031-s001.zip › metabolites-2605833-supplementary.pdf]

metabolites

Supporting Information

Food Monitoring: Limitations of Accelerated Storage to Predict Molecular Changes in Hazelnuts (*Corylus avellana* L.) under Realistic Conditions Using UPLC-ESI-IM-QTOF-MS

Henri Loesel [1], Navid Shakiba [1,2], Soeren Wenck [1], Phat Le Tan [1], Tim-Oliver Karstens [1], Marina Creydt [1], Stephan Seifert [1], Thomas Hackl [1,2] and Markus Fischer [1,*]

[1] Hamburg School of Food Science, Institute of Food Chemistry, University of Hamburg, Grindelallee 117, 20146 Hamburg, Germany; henri.loesel@uni-hamburg.de (H.L.); navid.shakiba@uni-hamburg.de (N.S.); soeren.wenck@uni-hamburg.de (S.W.); phat.le.tan@studium.uni-hamburg.de (P.L.T.); tim-oliver.karstens@studium.uni-hamburg.de (T.-O.K.); marina.creydt@uni-hamburg.de (M.C.); stephan.seifert@uni-hamburg.de (S.S.); thomas.hackl@uni-hamburg.de (T.H.)

[2] Institute of Organic Chemistry, University of Hamburg, Martin-Luther-King-Platz 6, 20146 Hamburg, Germany

* Correspondence: markus.fischer@uni-hamburg.de; Tel.: +49-40-42838-4359

Supplementary Figures

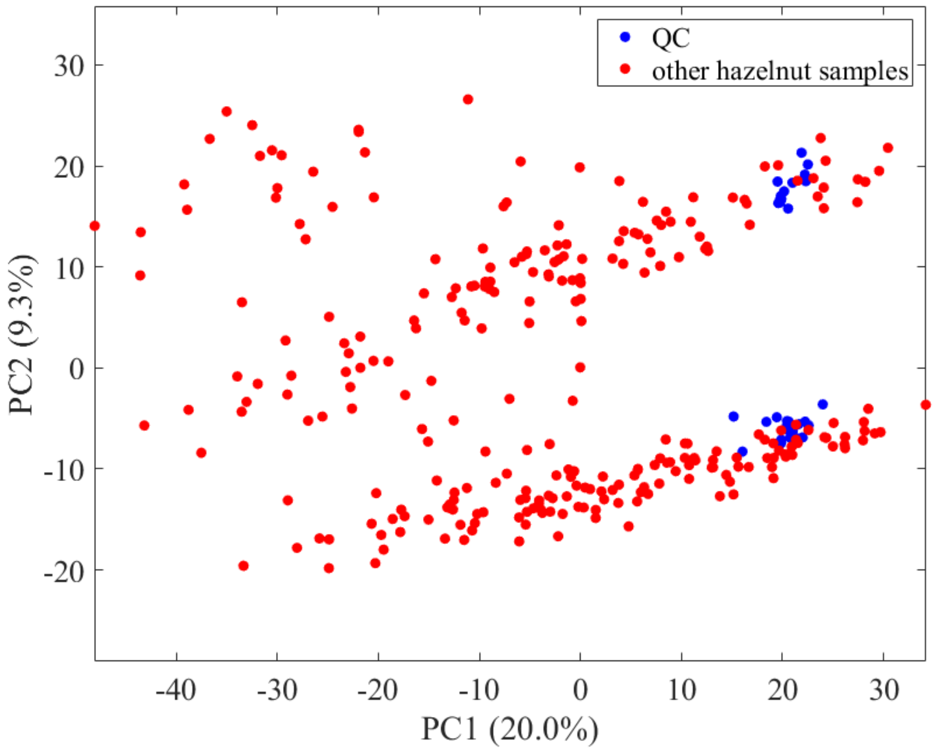

Figure S1. Scores of the principal component analysis depicted according to distinguish the quality control (QC) samples from the others. The data was previously vector normalized and autoscaled together regarding the two batches.

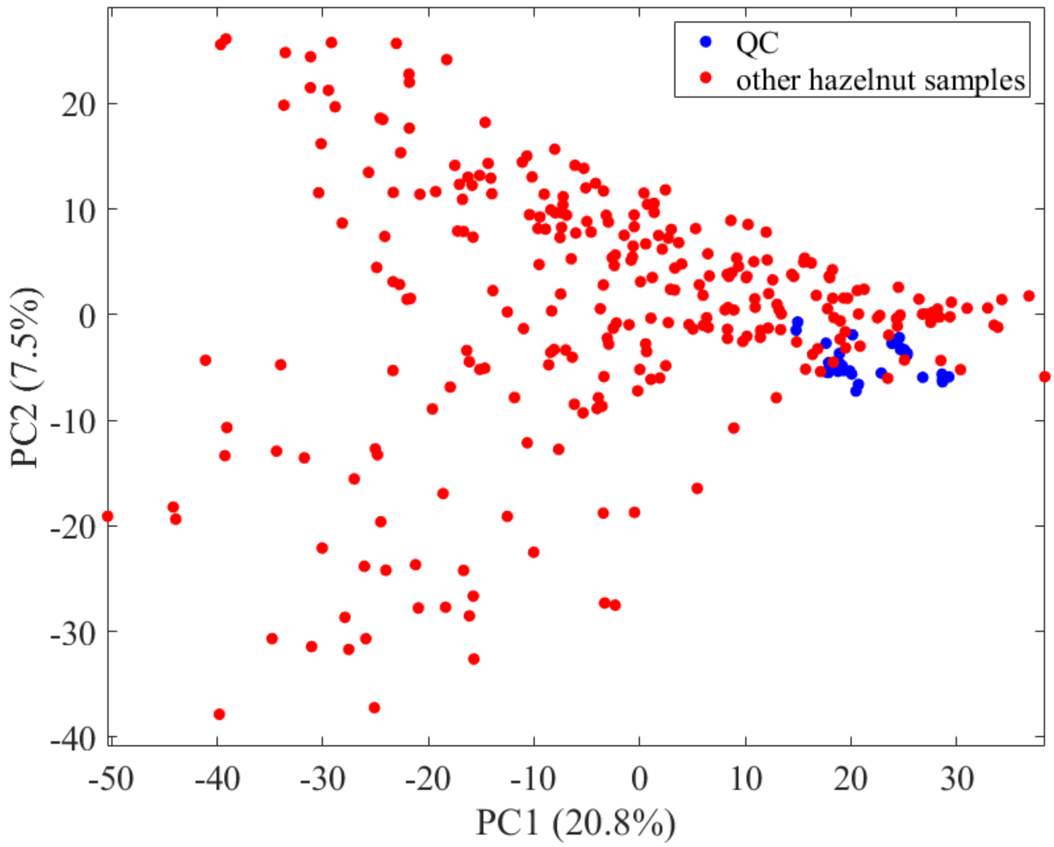

Figure S2. Scores of the principal component analysis depicted according to distinguish the quality control (QC) samples from the others. The data was previously vector normalized and autoscaled separately regarding the two batches..

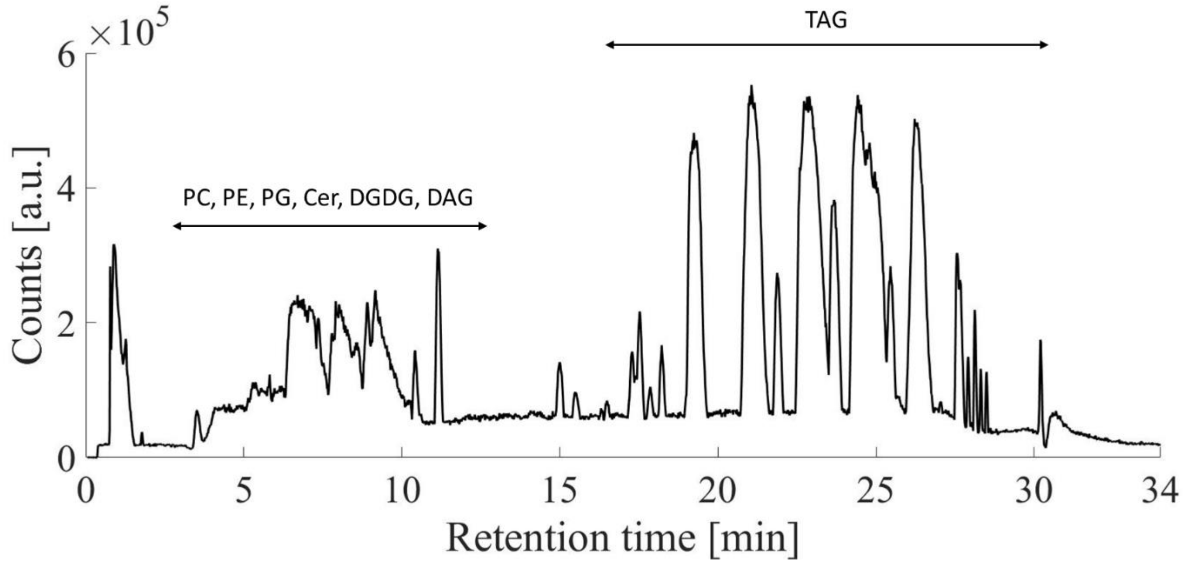

Figure S3. Exemplary TIC of a hazelnut sample with the following assignments of the main substance classes: PC: phosphocholine, PE: phosphoethanolamine, PG: phosphoglycerol, Cer: ceramide, DGDG: digalactosyldiacylglyceride, DAG: diacylglyceride, TAG: triacylglyceride.

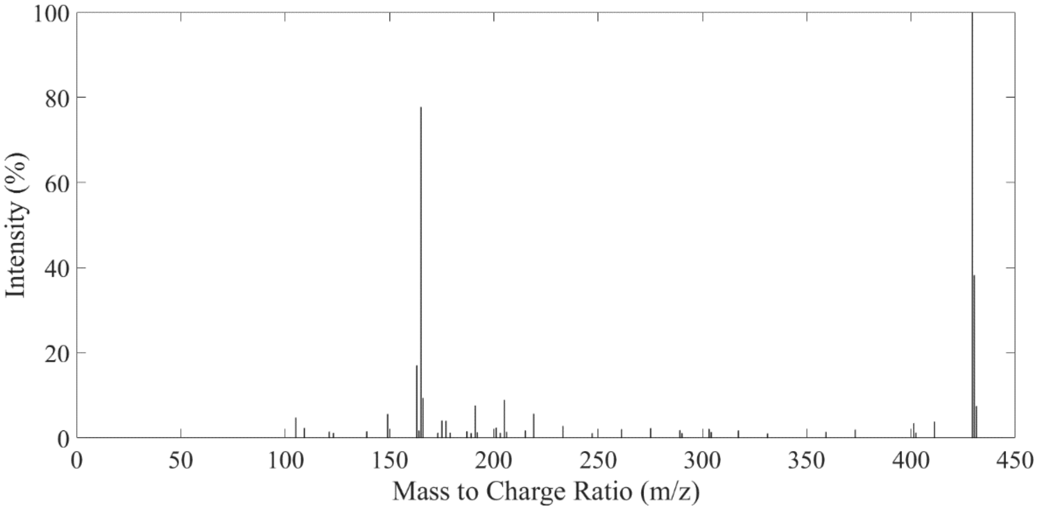

Figure S4. MS/MS spectrum of the metabolite, which was identified as α-tocomonenol. The collision energy was 10 eV.

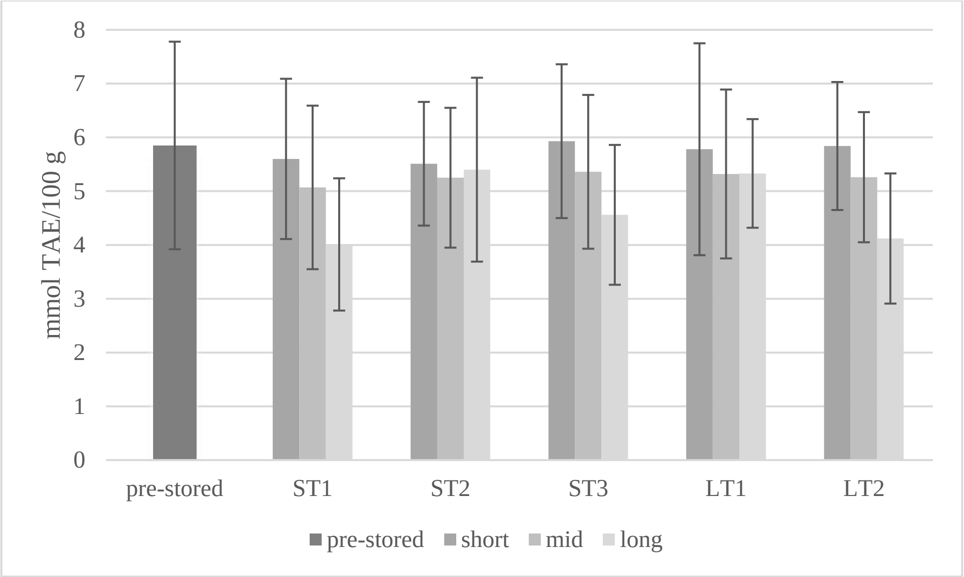

Figure S5. Bar graph of the results of the TEAC assay. The storage durations of accelerated short-term storage were summarized as follows: short (day 2, day 4, week 2), mid (week 6, week 10, week 12), and long (week 16, week 20, week 24). The storage periods of realistic long-term storage were summarized as follows: short (month 3, month 6), mid (month 9, month 12), and long (month 15, month 18).

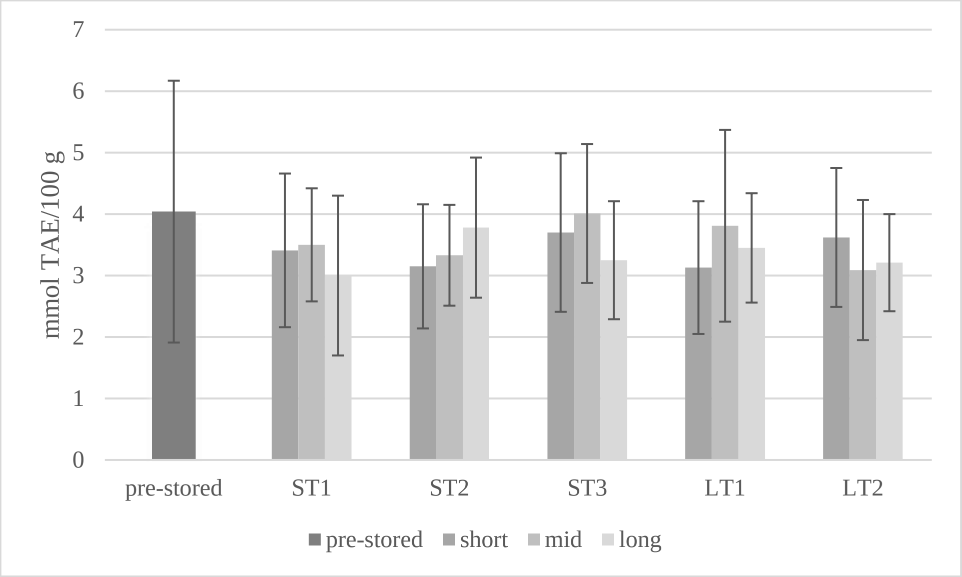

Figure S6. Bar graph of the results of the ORAC assay. The storage durations of accelerated short-term storage were summarized as follows: short (day 2, day 4, week 2), mid (week 6, week 10, week 12), and long (week 16, week 20, week 24). The storage periods of realistic long-term storage were summarized as follows: short (month 3, month 6), mid (month 9, month 12), and long (month 15, month 18).

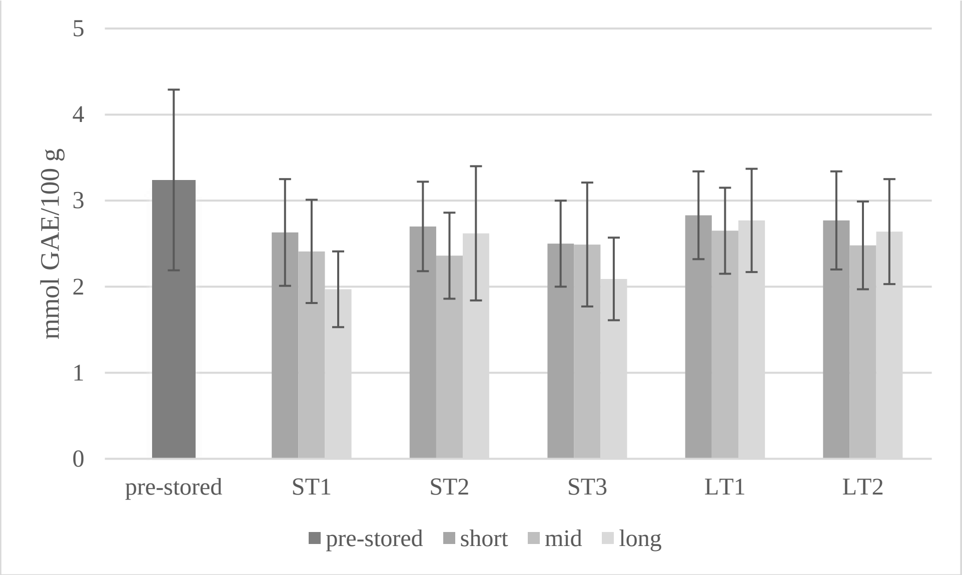

Figure S7. Bar graph of the results of the FOLIN-CIOCALTEU assay. The storage durations of accelerated short-term storage were summarized as follows: short (day 2, day 4, week 2), mid (week 6, week 10, week 12), and long (week 16, week 20, week 24). The storage periods of realistic long-term storage were summarized as follows: short (month 3, month 6), mid (month 9, month 12), and long (month 15, month 18).

Supplementary Tables

Table S1. Overview about the metadata of the stored hazelnut samples.

Sample-ID	Variety	Harvest year	Origin	Diameter [mm]
1	Mixture	2018	Turkey	11 – 13
2	Mixture	2019	Turkey	11 – 13
3	Tombul	2018	Turkey	13 – 15
4	Mixture	2019	Turkey	13 – 15

5	Mixture	2018	Turkey	>14
6	Mixture	2019	Turkey	>14

Table S2. Overview about the chosen storage durations.

ST1 – ST3 (weeks)	LT1 and LT2 (months)
2 days	3
4 days	6
2	9
4	12
6	15
8	18
10	
12	
16	
20	
24	

Table S3. Results of the classification of pre- and post-storage samples (post storage is defined by accelerated short-term storage ≥12 weeks and realistic long-term storage: ≥12 months; T_0: pre-storage, M_{12}: 12 months). The Sample-ID column shows which sample was not included in the training set and was subsequently classified using the trained model.

	**Sample-ID**	**1**	**2**	**3**	**4**	**5**	**6**
**ST1**	**Training accuracy [%]**	100	100	96	96	96	100
	**Validation [Amount of correct classified samples]**	5/5	5/5	5/5	5/5	5/5	5/5
	**Validation [Misclassified samples]**	/	/	/	/	/	/
**ST2**	**Training accuracy [%]**	72	80	80	80	80	84
	**Validation [Amount of correct classified samples]**	4/5	4/5	4/5	4/5	4/5	5/5
	**Validation [Misclassified samples]**	T_0	T_0	T_0	T_0	T_0	/
**ST3**	**Training accuracy [%]**	96	96	96	92	92	96
	**Validation [Amount of correct classified samples]**	4/5	5/5	5/5	5/5	5/5	5/5
	**Validation [Misclassified samples]**	T_0	/	/	/	/	/
**LT1**	**Training accuracy [%]**	75	75	80	80	75	80
	**Validation [Amount of correct classified samples]**	3/4	3/4	3/4	3/4	3/4	3/4
	**Validation [Misclassified samples]**	T_0	M_{12}	T_0	T_0	T_0	T_0
**LT2**	**Training accuracy [%]**	85	90	85	85	90	90
	**Validation [Amount of correct classified samples]**	3/4	4/4	3/4	4/4	3/4	4/4
	**Validation [Misclassified samples]**	T_0	/	T_0	/	T_0	/

Identification parameters

Phosphocholines were identified via the fragment at *m*/*z* 184 formed by cleavage of the polar head group. The corresponding [M+H-183]$^+$-fragment, on the other hand, showed only low intensity. The fatty acids were determined by difference calculation.

The characteristic fragment of phosphoethanolamines was the ion [M+H–141]$^+$, which was formed by the cleavage of the polar head group. The fragment formed was the diacylglyceride and subsequently cleaved off a fatty acid, which made it possible to obtain information about the fatty acids it contained.

Phosphoglycerols were identified by the fragment of the polar head group at *m*/*z* 195 , and fatty acids were determined by difference calculation.

Identification of ceramides based on fragment spectra was performed analogously to another study. The most intense signal in the fragmentation spectra showed a signal corresponding to the phytosphingosine moiety (*m*/*z* 326 or 324) formed after cleavage of the amide bond.

Fragment spectra of digalactosyldiacylglycerides revealed [M+Na–162]$^+$ fragments formed by cleavage of a galactosyl unit. Other fragments are formed when the monogalactosyl fragment cleaves off a fatty acid. In addition, fatty acids are cleaved directly from the molecular ion.

Oxidized lipids were identified by MS/MS spectra according to previous studies, where the mechanisms of formation and fragmentation were also described in detail. The epoxides were identified by the fragments of the diacylglycerides, which have an *m*/*z* +14 compared to the corresponding unoxidized diacylglyceride. This difference results from the addition of one oxygen atom and the abstraction of two hydrogen atoms.

Triacylglycerides were identified based on the fragments that result from the cleavage of a fatty acid and the neutral loss of this fatty acid. In addition, [RCOO + 58]$^+$ fragments were used for structure elucidation.

Table S4. Summary of identification parameters of marker compounds that are affected by storage.

Tentative metabolite	Storage condition	Sum formula	Ion	*m*/*z* (analytical)	*m*/*z* (calculated)	*m*/*z* difference [ppm]	RT [min]	DT [ms]	CCS-value [analytical, Å²]	CCS-value [LipidCCS database, Å²]	CCS-value [difference, %]	Main fragments [*m*/*z*]
**PC (0:0/0:0)**	ST1	$C_8H_{20}NO_6P$	+H	258.1104	258.1101	1.17	0.84	19.166	156.04	no entry	/	184.0733<br>104.1071
**α-tocomonoenol**	ST1	$C_{29}H_{48}O_2$	+H	429.3733	429.3727	1.38	8.47	26.790	214.62	no entry	/	205.1219<br>165.091
**PC (16:0/0:0)**	ST1	$C_{24}H_{50}NO_7P$	+H	496.3408	496.3398	2.09	3.41	28.721	229.28	230.5	0.53	184.0733<br>104.1072
**PC (18:2/0:0)**	ST1	$C_{26}H_{50}NO_7P$	+H	520.3408	520.398	1.99	2.65	28.468	226.96	229.7	1.19	184.0732<br>104.1073
**PC (18:1/0:0)**	ST1	$C_{26}H_{52}NO_7P$	+H	522.3566	522.3554	2.27	3.55	29.125	232.23	232.7	0.20	184.0735<br>104.1071
**Cer (d36:3)**	ST1	$C_{36}H_{67}NO_3$	+H	562.5194	562.5194	0.05	8.21	32.086	255.57	254.6 [Cer (d18:2/18:1)]	/	324.2905<br>306.2794<br>300.2902<br>282.2794

Tentative metabolite	Storage condition	Sum formula	Ion	*m/z* (analytical)	*m/z* (calculated)	*m/z* difference [ppm]	RT [min]	DT [ms]	CCS-value [analytical, Å²]	CCS-value [LipidCCS database, Å²]	CCS-value [difference, %]	Main fragments [*m/z*]
**Cer (d36:2)**	ST1	$C_{36}H_{69}NO_3$	+H	564.5361	564.535	1.91	9.45	32.266	257	258.6 [Cer (d18:1/18:1)]	/	326.3058 308.2956 300.2898 282.2798
**Cer (d38:4)**	ST1	$C_{38}H_{69}NO_3$	+H	588.5363	588.535	2.18	8.28	32.430	258.07	no entry	/	324.2903 306.2792
**Cer (d38:3)**	ST1	$C_{38}H_{71}NO_3$	+H	590.5519	590.5507	2.08	9.54	32.738	260.52	261.2 [Cer (d18:2/20:1)]	/	326.306 308.2954
**DAG (18:3/18:1/0:0)**	ST1	$C_{39}H_{68}O_5$	+H	617.5153	617.514	2.19	5.62	32.661	259.64	259.9	0.10	599.5038 339.2902 335.2592
**DAG (18:0/18:3/0:0)**	ST1, ST3	$C_{39}H_{70}O_5$	+H	619.5313	619.5296	2.75	6.94	33.523	266.53	264.6	-0.73	341.3056 335.2589
**TAG (18:1/18:1/2:0)**	ST3	$C_{41}H_{74}O_6$	$+NH_4$	680.5859	680.5824	5.33	11.1	34.361	272.7	no entry	/	663.5579 603.5361 381.3011 339.2898
**PE (18:2/18:2)**	ST1	$C_{41}H_{74}NO_8P$	+H	740.5242	740.5225	2.32	6.82	34.529	273.61	276.0	0.87	599.5044 337.2742
**PE (18:2/18:1)**	ST1	$C_{41}H_{76}NO_8P$	+H	742.5406	742.5381	3.33	7.72	34.875	276.36	278.1	0.63	601.5207 339.2893 337.2736
**PE (18:1/18:1)**	ST1	$C_{41}H_{78}NO_8P$	+H	744.5562	744.5538	3.25	8.84	35.185	278.82	281.5	0.95	603.5364 339.2911
**PG (36:5)**	ST1	$C_{42}H_{73}O_{10}P$	+H	769.5017	769.5014	0.38	4.03	35.320	279.74	281.6 [PG (18:2/18:3)]	/	597.4869 195.0026
**PC (36:4)**	ST1	$C_{44}H_{80}NO_8P$	+H	782.5729	782.5694	4.44	7.34	36.074	285.66	285.4 [PC (18:2/18:2)]	/	184.0731
**TAG (18:2/18:1/12:0)**	ST1	$C_{51}H_{92}O_6$	$+NH_4$	818.726	818.7259	3.48	18.82	38.493	304.72	305.8	0.35	601.5203 521.4572 519.4416
**TAG (18:2/18:2/16:1)**	ST1	$C_{55}H_{96}O_6$	$+NH_4$	870.757	870.7545	2.91	18.88	39.657	313.68	314.5	0.26	599.5049 573.4898
**TAG (18:2/16:1/20:5)**	ST1, ST3	$C_{57}H_{94}O_6$	+H	875.7133	875.7123	1.12	18.85	39.562	312.9	316.3	1.07	621.4848 595.4694 573.4892
**TAG (18:1/18:1/16:0)**	ST1	$C_{55}H_{102}O_6$	$+NH_4$	876.8046	876.8015	3.65	24.72	40.550	320.76	319.3	-0.46	603.5367 577.5215

Tentative metabolite	Storage condition	Sum formula	Ion	*m/z* (analytical)	*m/z* (calculated)	*m/z* difference [ppm]	RT [min]	DT [ms]	CCS-value [analytical, Å²]	CCS-value [LipidCCS database, Å²]	CCS-value [difference, %]	Main fragments [*m/z*]
**TAG (52:2) epoxidized**	ST3, LT2	$C_{55}H_{100}O_7$	$+NH_4$	890.7836	890.7807	3.29	17.26	40.527	320.49	no entry	/	873.7573<br>617.5151<br>603.537<br>591.5009<br>575.5053<br>339.29<br>313.274<br>491.3749<br>477.3944
**TAG (52:2) epoxidized**	ST1, ST3, LT2	$C_{55}H_{100}O_7$	$+NH_4$	890.7838	890.7807	3.52	16.26	40.546	320.64	no entry	/	855.7471<br>603.5366<br>591.5001<br>573.4895<br>499.4512
**TAG (52:1) epoxidized**	ST1, ST3	$C_{55}H_{102}O_7$	$+NH_4$	892.7996	892.7964	3.68	18.96	40.680	321.7	no entry	/	619.5325<br>601.5203<br>593.5161<br>577.5212<br>599.505<br>493.3899<br>477.3951<br>451.3794<br>339.2901<br>337.274<br>313.2742<br>263.2372
**TAG (18:3/18:2/18:2)**	ST1, ST3	$C_{57}H_{96}O_6$	$+NH_4$	894.7579	894.7545	3.86	17.44	40.064	316.79	318.5	0.54	877.7313<br>599.5028<br>597.4896<br>337.2726<br>335.2578
**TAG (54:5) epoxidized**	ST1, ST3	$C_{57}H_{98}O_7$	+H	895.7391	895.7385	0.63	16.24	40.276	318.47	no entry	/	615.5964<br>613.4791<br>293.2085
**TAG (18:2/18:2/18:2)**	ST1, ST3	$C_{57}H_{98}O_6$	$+NH_4$	896.7738	896.7702	4.14	19.19	40.316	318.79	319.3	0.16	879.7462<br>599.5052<br>337.274
**TAG (54:4) epoxidized**	ST1, ST3, LT2	$C_{57}H_{100}O_7$	+H	897.7564	897.7542	2.47	10.54	40.614	321.15	no entry	/	879.745<br>617.5155<br>615.4998<br>601.5194<br>339.2898

Tentative metabolite	Storage condition	Sum formula	Ion	*m/z* (analytical)	*m/z* (calculated)	*m/z* difference [ppm]	RT [min]	DT [ms]	CCS-value [analytical, Å²]	CCS-value [LipidCCS database, Å²]	CCS-value [difference, %]	Main fragments [*m/z*]
**TAG (18:1/18:2/18:2)**	ST1	$C_{57}H_{100}O_6$	$+NH_4$	898.7911	898.7858	6	20.99	40.674	321.62	320.7	-0.29	881.7639<br>601.5209<br>599.5057<br>339.2896<br>337.2741
**TAG (18:2/18:2/20:5)**	ST1	$C_{59}H_{96}O_6$	+H	901.7301	901.7338	2.37	19.27	40.187	317.73	321.3	1.11	621.4858<br>599.5038
**TAG (53:2) epoxidized**	ST1, ST3	$C_{56}H_{104}O_7$	$+NH_4$	906.8157	906.812	4.13	20.03	41.235	326.04	no entry	/	889.788<br>871.7766<br>619.5312<br>607.5302<br>601.5202<br>591.5351<br>493.3889<br>477.3949<br>339.2897<br>337.2738<br>327.2894
**TAG (52:0) epoxidized twice**	ST1, ST3, LT2	$C_{55}H_{102}O_8$	$+NH_4$	908.7942	908.7913	3.26	13.68	40.921	323.53	no entry	/	891.7645<br>873.7533<br>635.5248<br>617.5142<br>593.5144<br>575.5033<br>509.3834<br>493.3884<br>467.3729<br>451.3779<br>355.2843<br>337.2743<br>313.274
**TAG (54:6) epoxidized**	ST1	$C_{57}H_{96}O_7$	$+NH_4$	910.753	910.7494	4	12.54	40.195	317.74	no entry	/	613.484<br>599.5043<br>595.4729<br>491.3727<br>337.2733

Tentative metabolite	Storage condition	Sum formula	Ion	*m/z* (analytical)	*m/z* (calculated)	*m/z* difference [ppm]	RT [min]	DT [ms]	CCS-value [analytical, Å²]	CCS-value [LipidCCS database, Å²]	CCS-value [difference, %]	Main fragments [*m/z*]
**TAG (54:4) epoxidized**	ST1	$C_{57}H_{100}O_7$	$+NH_4$	914.7839	914.7807	3.53	15.95	40.756	322.19	no entry	/	897.7588<br>881.7638<br>617.5155<br>615.5007<br>601.5225<br>599.5039<br>597.4887<br>491.3743<br>339.2901<br>261.222
**TAG (54:3) epoxidized**	LT1, LT2	$C_{57}H_{102}O_7$	$+NH_4$	916.7984	916.7964	2.25	15.43	41.107	324.97	no entry	/	881.7641<br>617.5148<br>603.5364<br>599.506<br>339.2905<br>261.222
**TAG (54:3) epoxidized**	ST1, ST3	$C_{57}H_{102}O_7$	$+NH_4$	916.7992	916.7964	3.14	14.62	40.707	321.79	no entry	/	899.7704<br>881.7619<br>617.5129<br>601.5199<br>599.5034<br>339.2903<br>337.2727<br>261.2218
**TAG (54:3) epoxidized**	ST1, ST3	$C_{57}H_{102}O_7$	$+NH_4$	916.7997	916.7964	3.69	17.33	41.113	325.02	no entry	/	899.773<br>619.5305<br>617.5159<br>601.5212<br>599.5047<br>493.3891<br>491.374<br>477.3944<br>475.379
**TAG (54:2) epoxidized**	ST1, ST3	$C_{57}H_{104}O_7$	$+NH_4$	918.8159	918.812	4.29	19.11	41.498	328.07	no entry	/	901.7896<br>883.779<br>619.5324<br>603.5371<br>601.5197<br>493.3905<br>477.3956

Tentative metabolite	Storage condition	Sum formula	Ion	*m/z* (analytical)	*m/z* (calculated)	*m/z* difference [ppm]	RT [min]	DT [ms]	CCS-value [analytical, Å²]	CCS-value [LipidCCS database, Å²]	CCS-value [difference, %]	Main fragments [*m/z*]
**TAG (54:1) epoxidized**	ST1, ST3	$C_{57}H_{106}O_7$	$+NH_4$	920.8307	920.8277	3.34	21.07	41.840	330.78	no entry	/	903.8044<br>885.7931<br>621.546<br>619.5316<br>605.5529<br>601.5199<br>493.3895<br>477.3946
**TAG (54:5) epoxidized twice**	ST1	$C_{57}H_{96}O_8$	$+NH_4$	926.7466	926.7433	2.48	8.89	40.345	318.85	no entry	/	629.4783<br>615.4966<br>613.4813<br>597.4893<br>595.4705
**TAG (54:4) epoxidized twice**	ST1	$C_{57}H_{98}O_8$	$+NH_4$	928.7623	928.76	2.53	9.98	40.593	320.82	no entry	/	893.7264<br>631.4945<br>615.5003<br>613.4841<br>599.5054<br>491.3742<br>337.2746
**TAG (54:2) epoxidized twice**	ST1	$C_{57}H_{102}O_8$	$+NH_4$	932.7945	932.7913	3.5	12.49	41.238	325.92	no entry	/	915.7696<br>897.7572<br>633.5098<br>615.5<br>603.5363<br>491.3737<br>339.2884<br>277.2164
**TAG (54:1) epoxidized twice**	ST3	$C_{57}H_{104}O_8$	$+NH_4$	934.8096	934.8069	2.9	15.12	41.438	327.51	no entry	/	635.5244<br>619.5267<br>617.5128<br>603.5337<br>509.3846<br>493.3878<br>479.3731<br>339.2889
**TAG (54:2) epoxidized thrice**	ST1	$C_{57}H_{100}O_9$	$+NH_4$	946.7732	946.7706	2.84	7.74	40.878	323	no entry	/	929.7463<br>911.7361<br>633.5109<br>631.4954<br>617.5127<br>615.4996<br>599.5055<br>491.3748

Tentative metabolite	Storage condition	Sum formula	Ion	*m/z* (analytical)	*m/z* (calculated)	*m/z* difference [ppm]	RT [min]	DT [ms]	CCS-value [analytical, Å²]	CCS-value [LipidCCS database, Å²]	CCS-value [difference, %]	Main fragments [*m/z*]
**TAG (56:2) epoxidized**	ST1, ST3	$C_{59}H_{108}O_7$	$+NH_4$	946.8462	946.8433	3.09	20.57	42.088	332.61	no entry	/	647.5634<br>629.5537<br>603.5367<br>505.4268
**TAG (54:1) epoxidized thrice**	ST1, LT2	$C_{57}H_{102}O_9$	$+NH_4$	948.789	948.7862	3	8.51	41.130	324.99	no entry	/	633.511<br>617.5126<br>615.5011<br>599.504
**TAG (54:0) epoxidized thrice**	ST1, ST3, LT2	$C_{57}H_{104}O_9$	$+NH_4$	950.8034	950.8019	1.65	9.39	41.210	325.61	no entry	/	635.5253<br>933.776<br>617.5144<br>599.5033<br>509.3838<br>493.3894
**DGDG (18:2/18:2)**	ST1	$C_{51}H_{88}O_{15}$	+Na	963.6044	963.6015	3.04	6.03	39.374	310.96	no entry	/	801.551<br>683.3613<br>521.3087
**DGDG (18:1/18:2)**	ST1	$C_{51}H_{90}O_{15}$	+Na	965.6202	965.6172	3.19	6.74	39.614	312.86	no entry	/	803.5631<br>685.3791<br>683.3616<br>523.3236<br>521.3058
**DGDG (18:1/18:1)**	ST1	$C_{51}H_{92}O_{15}$	+Na	967.6361	967.6328	3.45	7.64	39.857	314.78	no entry	/	805.5795<br>685.3767<br>523.3228

Table S5: Results from the determination of antioxidant capacity (AOA) with the TEAC, ORAC, and FOLIN-CIOCALTEU (FC) assays. The storage durations of accelerated short-term storage were summarized as follows: short (day 2, day 4, week 2), mid (week 6, week 10, week 12), and long (week 16, week 20, week 24). The storage periods of realistic long-term storage were summarized as follows: short (month 3, month 6), mid (month 9, month 12), and long (month 15, month 18).

		TEAC (mmol TAE/100 g)			ORAC (mmol TAE/100 g)			FC (mmol GAE/100 g)		
Pre-stored	AOA	5.85 ± 1.93			4.04 ± 2.13			3.24 ± 1.05		
		short	mid	long	short	Mid	long	short	mid	long
ST1	AOA	5.60 ± 1.49	5.07 ± 1.52	4.01 ± 1.23	3.41 ± 1.25	3.50 ± 0.92	3.00 ± 1.30	2.63 ± 0.62	2.41 ± 0.60	1.97 ± 0.44
	p-value	7.43×10^{-01}	3.21×10^{-01}	1.19×10^{-02}	4.35×10^{-01}	4.54×10^{-01}	1.59×10^{-01}	9.43×10^{-02}	2.26×10^{-02}	3.24×10^{-04}
ST2	AOA	5.51 ± 1.15	5.25 ± 1.30	5.40 ± 1.71	3.15 ± 1.01	3.33 ± 0.82	3.78 ± 1.14	2.70 ± 0.52	2.36 ± 0.50	2.62 ± 0.78
	p-value	6.06×10^{-01}	3.98×10^{-01}	5.93×10^{-01}	2.34×10^{-01}	3.12×10^{-01}	7.01×10^{-01}	1.02×10^{-01}	1.01×10^{-02}	1.32×10^{-01}
ST3	AOA	5.93 ± 1.43	5.36 ± 1.43	4.56 ± 1.30	3.70 ± 1.29	4.01 ± 1.13	3.25 ± 0.96	2.50 ± 0.50	2.49 ± 0.72	2.09 ± 0.48
	p-value	9.10×10^{-01}	5.17×10^{-01}	7.77×10^{-02}	6.69×10^{-01}	9.64×10^{-01}	2.13×10^{-01}	2.73×10^{-02}	6.24×10^{-02}	1.11×10^{-03}
LT1	AOA	5.78 ± 1.97	5.32 ± 1.57	5.33 ± 1.01	3.13 ± 1.08	3.81 ± 1.56	3.45 ± 0.89	2.83 ± 0.51	2.65 ± 0.50	2.77 ± 0.60
	p-value	9.50×10^{-01}	5.42×10^{-01}	4.62×10^{-01}	2.37×10^{-01}	4.11×10^{-01}	7.94×10^{-01}	2.67×10^{-01}	1.18×10^{-01}	2.39×10^{-01}
LT2	AOA	5.84 ± 1.19	5.26 ± 1.21	4.12 ± 1.21	3.62 ± 1.13	3.09 ± 1.14	3.21 ± 0.79	2.77 ± 0.57	2.48 ± 0.51	2.64 ± 0.61
	p-value	9.89×10^{-01}	4.39×10^{-01}	3.26×10^{-02}	5.80×10^{-01}	2.28×10^{-01}	2.35×10^{-01}	2.24×10^{-01}	5.09×10^{-02}	1.40×10^{-01}
